# A deterministic approach for protecting privacy in sensitive personal data

**DOI:** 10.1186/s12911-022-01754-4

**Published:** 2022-01-28

**Authors:** Demetris Avraam, Elinor Jones, Paul Burton

**Affiliations:** 1 Population Health Sciences Institute, Newcastle University, Newcastle, UK; 2 Department of Public Health, University of Copenhagen, Copenhagen, Denmark; 3 Department of Statistical Science, University College London, London, UK

**Keywords:** Data privacy, Deterministic anonymisation, Disclosure risk, Information loss, *k* nearest neighbours

## Abstract

**Background:**

Data privacy is one of the biggest challenges for any organisation which processes personal data, especially in the area of medical research where data include sensitive information about patients and study participants. Sharing of data is therefore problematic, which is at odds with the principle of open data that is so important to the advancement of society and science. Several statistical methods and computational tools have been developed to help data custodians and analysts overcome this challenge.

**Methods:**

In this paper, we propose a new deterministic approach for anonymising personal data. The method stratifies the underlying data by the categorical variables and re-distributes the continuous variables through a *k* nearest neighbours based algorithm.

**Results:**

We demonstrate the use of the deterministic anonymisation on real data, including data from a sample of Titanic passengers, and data from participants in the 1958 Birth Cohort.

**Conclusions:**

The proposed procedure makes data re-identification difficult while minimising the loss of utility (by preserving the spatial properties of the underlying data); the latter means that informative statistical analysis can still be conducted.

## Introduction

Protection of privacy in personal data is a key societal concern with implications for scientific, commercial and government communities. Recent legislation such as the EU General Data Protection Regulation highlights this need to handle sensitive personal data carefully. At the same time, however, modern science emphasises the importance of transparency and reproducibility of scientific research [1]; indeed, open data and open science is arguably one of the biggest gains of the data revolution [2]. This has resulted in increasing demands from funders and publishers for the release of data, among other things, to accompany grant proposals and journal articles (see for example the proposition from the International Committee of Medical Journal Editors [3]).

This potential conflict between data sharing and protection of sensitive data means that data custodians must use disclosure controls and anonymisation methodologies to maintain individual confidentiality when sharing data. This is especially so in disciplines where collected data include sensitive information protected by ethico-legal regulations. Achieving anonymity is complicated however, as granular data can be mapped back to the individual who provided them relatively easily. Such demands and requirements have therefore led to the development of a range of data sharing solutions, commonly achieving privacy protection through pseudonymisation, encryption or confusion-based approaches [4–8]. However, no single solution can fully protect a dataset and often combinations of methods and multifaceted systems are essential.

### Existing methods for sharing sensitive data

A number of approaches to sharing sensitive data are in existence. Some of these are theoretically straightforward in their approach, for example simple adjustments of direct identifiers (such as names and other forms of direct identifiers), or trusted data sharing within secure policy and procedural frameworks such as the Data Safe Havens and the Trusted Research Environments [9, 10]. Even in the latter scenario, the stripping of direct identifiers is often required and constitutes good practice.

However, other more technical or statistical solutions may be considered as alternatives. At one end of the spectrum, ‘black box’ computing tools like DataSHIELD [11–13] and ViPAR [14] can be used to control accessibility and allow secure federated data analysis. Such innovative tools mitigate disclosure risk while preserving data utility and allowing statistical analysis to be performed on the original data. Here the term ‘original data’ refers to the real data for analysis but they may already have been pre-processed (e.g., cleaned, harmonised, transformed, etc.) and/or pseudonymised (i.e., direct identifiers removed or disguised) before data storage. Black box approaches often restrict the analysis to a limited range of statistical methods and on specific outputs that the analyst can receive and publish (often non-disclosive low-dimensional summary statistics are returned to the analyst).

At the other end of the spectrum, simulated synthetic data [15] can provide full protection of privacy and eliminate any re-identification risk. However, synthetic data often do not capture the higher-order statistical properties of the underlying data, as the synthesis relies on simulation of entirely new values from a simplified statistical model. The quality of synthetic data depends strongly on the ability of the selected models to capture important relationships found in the original data. Thus the loss of information in simulations of synthetic datasets can be significant but these data can be very useful for designing research studies, developing software tools and methodologies, and for training purposes (see for example, the generation of synthetic data for the development of a proof-of-concept biomedical data exploration and visualisation tool in Virtual Reality [16]).

However, analysis of anonymised data can often be preferable to working via a tightly-controlled disclosure-mitigating black-box or to the analysis of synthetic data. In contrast to the former, the analyst can then use any statistical methods he/she chooses and unlike the latter the analytic data preserves all of the underlying statistical properties of the original data. This has given rise to a number of privacy protection techniques that do not require black boxes or the creation of synthetic data. As well as suppressing direct identifiers (pseudonymisation), examples of such extended techniques include (1) the control of direct identifiers, unique combinations of records, outlier values, or small cell counts, (2) the aggregation of granular detailed data to wider groups or categories, (3) the rounding of numerical values to numbers with fewer significant figures, (4) the perturbation of data with addition or multiplication of random stochastic noise, etc. Many of these focus on minimising disclosure risk and utility loss.

Strategically, each method or tool for disclosure control aims to reduce the risk of re-identification and to minimise the loss of data utility. However, no single solution is adequate to fully protect the data and even the most complex and sophisticated of solutions never offer informative data with zero risk of disclosure. The choice of the optimal approach is often assessed in terms of retaining the desired statistical properties of the data and is therefore heavily context-dependent [17]. Consequently, the merits of each approach should be carefully considered for any given dataset.

For example, the *k*-anonymisation process [18], which falls into categories (1)–(3) above, preserves privacy by reducing the granularity of the data by combining the suppression of records and aggregation of variables. It cannot be used in cases where unique patterns or outliers (e.g., a rare disease indicator) are essential for the statistical analysis because the change or suppression of this information can lead to an incorrect conclusion (e.g., an incorrect medical diagnosis or decision). Other weaknesses of *k*-anonymisation are the biases and skewing effects that it introduces to the data [19], and the high amount of information loss when it is applied to high-dimensional datasets [20].

Meanwhile probabilistic anonymisation, which falls into category (4) above, is based on randomisation and therefore each data custodian can choose the level of noise used to perturb their data. There are a number of modelling techniques that can ‘remove’ the effect of added noise from a regression outcome and recover the true model parameters [21–23]; this is, of course, a desirable property of the procedure. However, care needs to be taken with this technique: the release of many ‘noisy’ datasets generated from the same input dataset, but using a different random number generator seed, can give rise to inferences for the observed underlying data (i.e., the average of many noisy datasets converges to the expected value of the input).

### Contribution of this paper

In this paper we present a new deterministic procedure for protecting privacy in personal data. The proposed method obscures the micro-level information from a set of personal data while retaining the spatial statistical properties of the numerical variables. In contrast to *k*-anonymisation, this approach does not reduce the granularity of the data. Furthermore, it does not depend on randomisation and so the same output will always be produced given particular input parameters (in contrast to probabilistic anonymisation). This procedure has been recently used for the generation of privacy-preserving data visualisations [24].

The paper is structured as follows. In the “Methodology” section we introduce the deterministic anonymisation procedure and we explicitly describe its five basic steps. We also outline the metrics that we use to measure the *disclosure risk* of the anonymised data and the *information loss* generated due to the data perturbation during anonymisation. In the “Applications” section we apply the method to two real datasets: (1) a publicly available sample of data about passengers on the Titanic; and (2) a set of data from participants in the National Child Development Study [25]. This second set of data had previously been created and harmonised for use—by a large research team including us—in the analysis of “healthy obesity” as part of the BioSHaRE-EU project [26]. As a second part of the same project, we also used these same data to support the methodological development of DataSHIELD as a black-box solution to disclosure control [11]. In the “Applications” section we also conduct a sensitivity analysis to examine the effect of the number of nearest neighbours used in the procedure (parameter *k*) and the effect of the sample size to the information loss and disclosure risk of deterministically anonymised data. Finally, we discuss the strengths and limitations of the proposed procedure.

## Methodology

### Anonymisation procedure

The anonymisation method we propose in this paper stratifies the underlying data by all combinations of the levels in the categorical variables and then re-distributes the continuous variables by replacing each record with the centroid of itself and its $k-1$ nearest neighbours for some specified *k*. It is important to note here that our procedure means that the categorical variables remain untouched by the anonymisation process; we ensure that this is safe in step (2) below. It is only the continuous data that change during the proposed anonymisation process.

The procedure consists of five basic steps:

1. Standardisation;
2. Stratification;
3. Location of *k* nearest neighbours;
4. Estimation of centroids;
5. Scaling and re-centralisation of the data.

These steps are detailed below, but before their application we check that the dataset of individual records includes both continuous and categorical variables. If the given dataset includes only continuous variables, then step (2)—stratification—does not take place and the entire dataset is considered as a unique subset. Note that we are assuming that there is at least one continuous variable in the dataset.

#### Step (1): standardisation

We standardise each continuous variable using z-score transformation such that each variable is centralised to zero mean and scaled to one standard deviation.

#### Step (2): stratification

We stratify the data in relation to all possible combinations of the levels of categorical variables: each subgroup is now homogeneous in terms of the categorical data. For example, if a dataset includes three categorical variables where two of them are binary and the third has four categories, then the dataset is classified into $2 \times 2 \times 4=16$ different subsets.

To make re-identification harder, we ensure that the data are *k*-*anonymous* in each subset (i.e., each possible combination of levels of the categorical variables exists in the data at least *k* times). If *k*-anonymity does not occur in one or more subsets (i.e., at least one subset has less than *k* observations), then we apply existing methods to achieve the *k*-anonymity. This may include generalisation (i.e., individual values are aggregated in broader categories) and/or suppression (i.e., unique or rare patterns of individual records are removed from the dataset).

Note that the *k* used here is the same as the value used for the location of each *k* nearest neighbours (see the next step of the algorithm). The choice of *k* is open here, but is most likely that when choosing *k* large enough, the risk of disclosure is sufficiently small. However, choosing a very large *k* will result in substantive loss of information: for example, choosing *k* equal to the size of the smallest stratum will mean that each continuous variable for observations in this stratum will all be mapped to the same value in the next step.

#### Step (3): location of each observation’s $k$-1 nearest neighbours

This step of the method locates the $k-1$ nearest neighbours of each observation, for which we use the so-called *k*-NN (*k* nearest neighbours) algorithm. This procedure is based on a technique in machine learning and is widely used in several disciplines such as computational geometry [27], diagnostic medicine [28], cryptography [29], data mining [30], and as here, in solutions for data privacy [31].

The *k*-NN algorithm is implemented separately in each stratum as formed in the previous step. The nearest neighbours are defined as the data points with minimum distances from each point of interest. Here we use the Euclidean distance as the distance metric between each two standardised coordinate vectors.

#### Step (4): estimation of centroids

When the $k-1$ nearest neighbours of each data point are identified, the algorithm computes the coordinates of the centroid of each *k* data points (i.e., the $k-1$ neighbours and the point of interest). The coordinates of each centroid are calculated as the average of the coordinates of the nearest neighbours in each dimension separately. For example, in a 2D space the x-coordinate of the centroid is the mean of the x-coordinates of the *k* nearest data points and the y-coordinate of the centroid is the mean of the y-coordinates accordingly.

The continuous values of each individual data point are then replaced by the coordinates of the corresponding centroid. These coordinates are then pooled together into a single data frame, which includes values for all strata (i.e., a reverse of the stratification process). The contents of this new data frame are the *masked* data.

#### Step (5): scaling and re-centralisation

We apply scaling and shift to relocate the masked data back to the observed scale and location of the original data using an inverse of the z-score transformations applied in step (1). We multiply each continuous variable from the masked dataset (i.e., the centroids) with the ratio between the standard deviation of the original variable and the standard deviation of the masked variable. Adding the observed means of the original data to each continuous variable from the masked dataset completes the re-centralisation.

Scaling is desirable because the third and the fourth steps typically shrink the overall variability of the data. However, it should be noted that when the scaling factor is sufficiently large, outliers or influential points may be displaced out of the convex hull of the original data. In particular, the masked data are not restricted to the space occupied by the original data.

### Risk-utility evaluation

Two key features should be addressed before releasing any anonymised data. Firstly, what is the risk of disclosure using the anonymised data? This will determine if the (anonymised) dataset is secure enough to be released. Secondly, how much information in the data was lost during the anonymisation process? That is, what is the utility loss? Of course, there is a trade-off between the risk of disclosure and utility loss: methods that lower the risk of disclosure tend to increase utility loss.

Utility loss and disclosure risk evaluation is highly context-specific and no metric can act as a catch-all for either concern. In this paper we use three metrics to evaluate information loss, while a measure based on robust Mahalanobis distance will serve as a metric for disclosure risk.

#### Metrics for utility loss

The three metrics that we employ, look at different aspects of what it means to lose information due to the perturbation of the original values during anonymisation.

The first metric is a global metric that compares the entire anonymised dataset with the original data. We use a summary statistic of the propensity scores as the global measure of data utility of the anonymised data as proposed by Woo et al. [32]. To calculate the propensity scores, we first merge the original and the anonymised datasets vertically and we add a binary indicator which is equal to one for all records from the anonymised dataset and zero for all records from the original dataset. Then we estimate the propensity score which is the probability of a record being in the anonymised dataset. The estimated propensity score ${\hat{p}}_{i}$ of each record *i* is the predicted probability from a logistic model of the generated binary indicator regressed on all the variables in the merged dataset.

We then compare the distributions of the propensity scores in the original and masked data. If the two distributions are similar, we conclude that the data utility loss due to anonymisation is small. This global information loss [32] is given by

$$\begin{aligned} U = \frac{1}{N}\sum ^{N}_{i=1} \left({\hat{p}}_{i}-c\right)^2 \, , \end{aligned}$$

where *N* is the total number of rows in the merged dataset, ${\hat{p}}_{i}$ is the estimated propensity score for unit *i*, and *c* is the proportion of anonymised data in the merged dataset (in our procedure $c=0.5$ as the original and anonymised datasets have the same number of observations). When the anonymised and original datasets are identical (no loss of utility, and we cannot distinguish between the two datasets), the distributions of their propensity scores are identical and *U* is equal to zero (i.e., no utility loss). If on the other hand the anonymised dataset can be entirely identified through the logistic regression above, then we have lost all utility and *U* is 1/4. Note, however, that this metric does not take into consideration the natural pairing of the original data values and their corresponding centroids. This metric may therefore not capture the more nuanced utility loss.

The second metric that we use to measure information loss is variable-specific, and unlike the first metric, takes into account the pairing between the real and anonymised observations. For each continuous variable we calculate the Euclidean distance between each original value and its corresponding anonymised value. We then estimate the mean of the squares of the Euclidean distances and divide it by the variance of the original variable. This standardised metric of the Euclidean distances of a variable ${\varvec{x}}$ is given by

$$\begin{aligned} \delta _{{\varvec{x}}} =\frac{\frac{1}{n} \sum ^{n}_{i=1} \big (x_i^{or} - x_i^{an}\big )^2}{\text {Var}({\varvec{x}}^{or})}, \end{aligned}$$

where ${\varvec{x}}^{or}=(x_1^{or}, \ldots , x_n^{or})$ is the variable in the original dataset, ${\varvec{x}}^{an}=(x_1^{an}, \ldots , x_n^{an})$ is the corresponding variable in the anonymised dataset (both presented here in vector formats), and *n* is the length of the variables. This fraction indicates how much of the variability of the anonymised variable is introduced through the anonymisation. For example, if $\delta _{{\varvec{x}}}$ = 0.4, we conclude that 60% of the variability of ${\varvec{x}}^{an}$ is inherited from the observed variability of ${\varvec{x}}^{or}$ while 40% is introduced due to anonymisation.

The third metric is an analysis-specific measure of utility loss. To make the analysis-specific comparison we must assume an intended analysis for the data; here we suppose that a regression model is required. The proposed regression model is applied to both datasets separately. A standardised difference between the estimate of the regression coefficients derived from the original data and the estimates derived from the anonymised data is then computed. The standardised difference is calculated as

$$\begin{aligned} d_{{\hat{\beta }}}=\frac{\vert {\hat{\beta }}_{or}-{\hat{\beta }}_{an}\vert }{ \text {SE}({\hat{\beta }}_{or})}\, , \end{aligned}$$

where ${\hat{\beta }}_{or}$ and ${\hat{\beta }}_{an}$ are the coefficients of the same model estimated from the original and the anonymised data respectively and $\text {SE}({\hat{\beta }}_{or})$ is the estimated standard error of the coefficients from the original data [33]. This metric can be thought of as a standardised bias between the estimates from the original and anonymised data. Checking for overlap between the confidence intervals of the regression coefficients from the original and anonymised data has also been suggested [33, 34].

#### Metrics for disclosure risk

A risk assessment is based on the specific data disclosure risk scenario that pertains and can be tackled from the point of view of the individual level data (either to assess for identity or attribute disclosure) or from the point of view of the global level data (risk related to the entire dataset or risk related to the protection method).

For the evaluation of the disclosure risk of anonymised data we use a robust Mahalanobis distance-based metric [35], which is estimated by the *dRiskRMD* function from the *sdcMicro* package in R [36]. This approach counts the number of ‘risky’ observations associated with higher probability of re-identification. Risky observations are those that have not been sufficiently perturbed and are located in the same vicinity as their original values (i.e., in very close proximity, and below an accepted threshold). In particular, it takes into account outliers and rare combinations of variables; these are usually at higher risk of identification in comparison to, for example, observations closer to centre of the mass of a dataset.

The algorithm that estimates the disclosure risk first calculates the robust Mahalanobis distance between each observation in the original dataset (a vector) and the mean vector of the original data. It then generates an interval around each (multivariate) original observation, where the length of the interval is defined by its squared robust Mahalanobis distance scaled by a constant, $w_1$. These intervals create (possibly multidimensional) boxes around each observation. This scalar is a weight for adjusting the influence of the robust Mahalanobis distance and is chosen by the data custodian. Using this algorithm, we estimate two risks of disclosure.

The first risk of disclosure, *risk1*, is defined as the proportion of the anonymised values that fall into the intervals of their corresponding original values. Observations that fall within the intervals are considered potentially unsafe. The second metric, *risk2*, looks further at each observation flagged by *risk1*: it checks whether there are *m* other observations from the anonymised dataset very close to it, and computes the proportion of observations that are both at risk (according to *risk1*) and do not have *m* other observations close by. Note that *risk2* will always therefore be at least as small as *risk1*. We use Euclidean distances here, and ‘close’ means being within some scalar $w_2$ of the observation. The idea here is that if more than *m* points are ‘close’ to the observation then we consider this observation as safe. The parameters *m* and $w_2$, are chosen by the data custodian.

## Applications

### The Titanic passengers’ data

Our first application illustrates the deterministic procedure through the anonymisation of a sample of Titanic passengers data. The dataset was obtained from Kaggle (https://www.kaggle.com/c/titanic/data) and includes 12 variables for 891 Titanic passengers. Our aim is to model the survival of passengers onboard the Titanic. We exclude three variables that are not considered important in predicting survival: ticket and the cabin numbers, and the trichotomous indicator of where the passengers embarked on the ship (Cherbourg, Southampton, or Queenstown).

The remaining nine variables are listed in Table 1 and include a unique ID for each passenger; a binary indicator showing if the passenger survived (1) or died (0); the passenger’s class (1st, 2nd, 3rd); the passenger’s name; sex; age; the number of siblings and spouses aboard the Titanic (*SibSp*); the number of parents and children aboard the Titanic (*ParCh*); and the fare paid for the ticket. In total there are therefore five categorical variables and a further two continuous variables.

**Table 1:** An example of four records from the Titanic passengers data

Passenger Id	Survived	Pclass	Name	Sex	Age	SibSp	ParCh	Fare
1	0	3	Braund, Mr. Owen Harris	Male	22	1	0	7.2500
2	1	1	Cumings, Mrs. John Bradley	Female	38	1	0	71.2833
3	1	3	Heikkinen, Miss. Laina	Female	26	0	0	7.9250
$\vdots$	$\vdots$	$\vdots$	$\vdots$	$\vdots$	$\vdots$	$\vdots$	$\vdots$	$\vdots$
891	0	3	Dooley, Mr. Patrick	Male	32	0	0	7.750

We apply the deterministic procedure described in the “Methodology” section to anonymise the data and then apply a logistic regression model to predict the survival of the Titanic passengers in the given sample.

#### Anonymisation

In a realistic risk assessment scenario, the data custodian should first pseudonymise the data by replacing the passengers names (which is the only direct identifier) with non-identifiable pseudo codes. For simplicity, we assume that the generated pseudo codes are the contents of the *PassengerId* variable already included in the data. The features that are left for the analysis are the variables *Sex*, *Age*, *Pclass*, *SibSp*, *ParCh*, *Fare* and the outcome variable *Survived*. From those variables, *Age* includes 177 missing values which are filled by the median age of the rest 714 passengers. We then convert the two discrete numerical variables—*SibSp* and *ParCh*—to a binary indicator (*Family*) denoting whether each passenger has any family members aboard the ship or not. Excluding any names and/or passenger identifiers, we now have four categorical variables (*Survived*, *Class*, *Sex* and *Family*) and two numeric variables (*Age* and *Fare*).

**Fig. 1:**
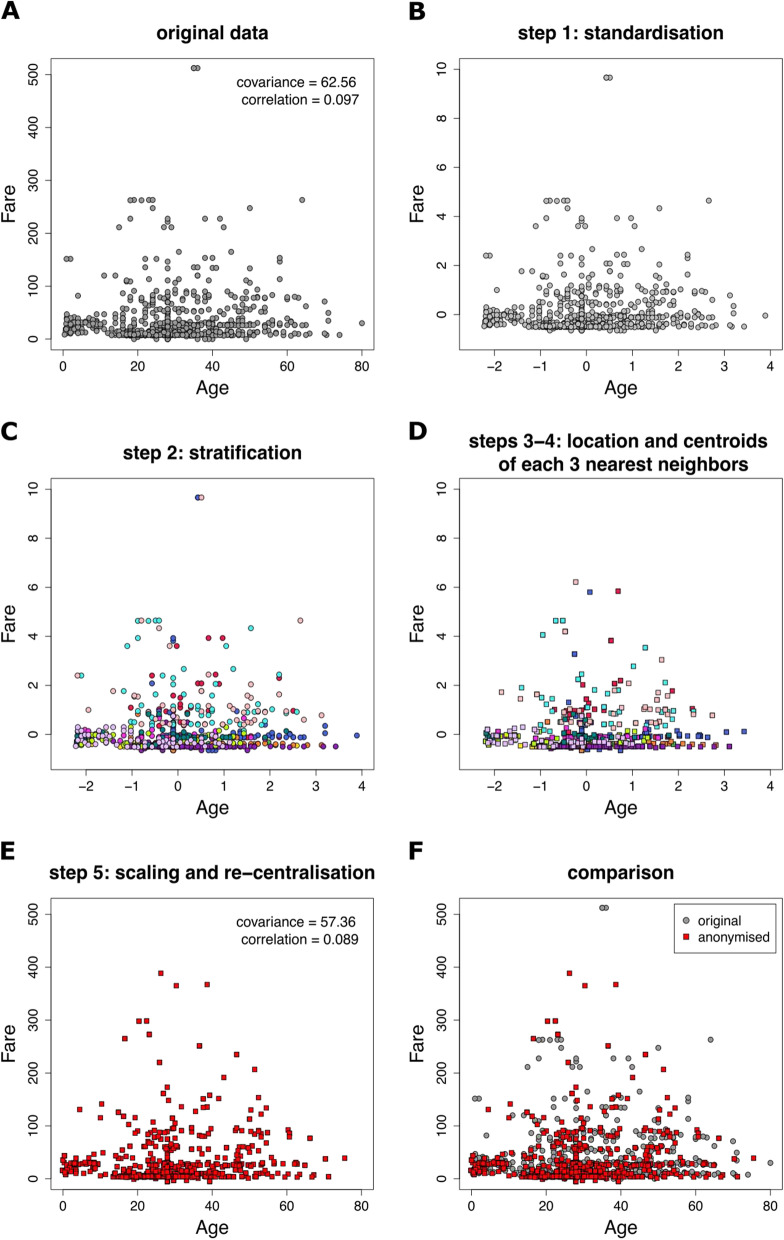
Illustration of the steps of the anonymisation procedure applied to Titanic passengers data. **A** Original continuous variables, **B** the variables after standardisation, **C** stratification of the variables in the 12 distinct strata, **D** the centroids of each 3 nearest neighbours, **E** scaled centroids and re-centralised back to the observed means (that are the anonymised data), **F** a comparison between the original and the anonymised variables

After preparing the data we follow the steps of the anonymisation process. Given that there are only two continuous variables, and it is only the continuous variables that go through the anonymisation procedure, the five steps of the anonymisation procedure as applied in the Titanic passengers data are visualised in Fig. 1. In particular, step (2) of the procedure checks if *k*-anonymity holds for the categorical variables for some *k*. Table 2 shows the 12 distinct strata that can be formed by all combinations of categories of the variables *Pclass*, *Sex* and *Family*. From the Frequency column we can see that the minimum stratum has 32 observations which means that 32-anonymity is applied. We will use *k* = 3 for the rest of this example, and given that 32-anonymity applies then trivially the data satisfy the requirement of being 3-anonymous.

Table 2 also presents the survival ratio that is the number of survived passengers over the total number of passengers in each stratum. By assuming that the *Survived* indicator is a sensitive attribute of the Titanic passengers we observe that there is significant uncertainty on disclosing this information for any particular individual (i.e., in no strata did all passengers survive or all died). This makes any attempt at inferential disclosure harder. For example, even if we know that a female was travelling in the first class of the Titanic with no family members, then we can only infer that the female died with probability 1/34.

**Table 2:** Frequencies of all possible combinations in the levels of categorical variables *Pclass*, *Sex* and *Family*

Pclass	Sex	Family	Frequency	Survival ratio
1	Female	0	34	33/34
2	Female	0	32	29/32
3	Female	0	60	37/60
1	Male	0	75	25/75
2	Male	0	72	7/72
3	Male	0	264	32/264
1	Female	1	60	58/60
2	Female	1	44	41/44
3	Female	1	84	35/84
1	Male	1	47	20/47
2	Male	1	36	10/36
3	Male	1	83	15/83

The table also shows the survival ratio that is the number of survived passengers over the total number of passengers in each stratum

With confirmation that the categorical variables in the dataset are 3-anonymous after stratification, we apply the anonymisation procedure to mask the real values of the continuous variables *Age* and *Fare*.

#### Disclosure risk and utility loss

Following the anonymisation procedure we calculate the utility loss and disclosure risk of the anonymised continuous data. The disclosure risks as measured by the robust Mahalanobis distance-based approach, with $w_1$ = 0.01 and $w_2$ = 0.05, are *risk1* = 0.0426 and *risk2* = 0.009, which are indicators of the percentage of sensitive observations. In particular, the *risk1* measure indicates that around 4% of the observations are ‘risky’—38 observations in total—in that they fall into the defined intervals around their corresponding original value. The *risk2* metric indicates that only 0.9% of the observations—8 observations in total—are actually ‘unsafe’ in that they fall into the defined interval around their original value and no other observations are in close proximity. Note that here we use the default values set in *dRiskRMD* function for the parameters $w_1$ and $w_2$ and we further investigate the impact of their variation in the next section.

**Fig. 2﻿:**
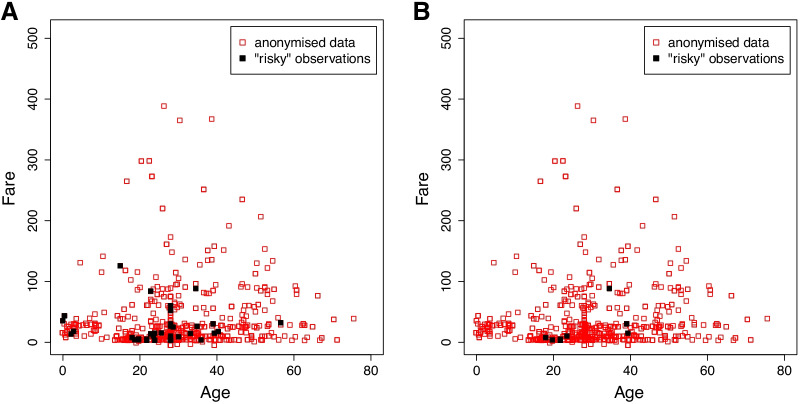
Risky observations according to the robust Mahalanobis distance-based metric. **A** 38 observations are considered as risky according to *risk1*, **B** 8 observations of those are considered as unsafe according to *risk2*

We can view these observations to better understand the nature of the potential disclosure, as shown in Fig. 2. It is observed that the points that are considered risky are points within the mass of the data points while extreme points (e.g., *Fare*>300) are unexpectedly considered ‘safe’. This happens because points in the center of the mass of the dataset tend to be replaced by centroids with high proximity to them due to the deteministic anonymisation, while extreme points such as outliers tend to be replaced by more distant centroids.

**Fig. 3:**
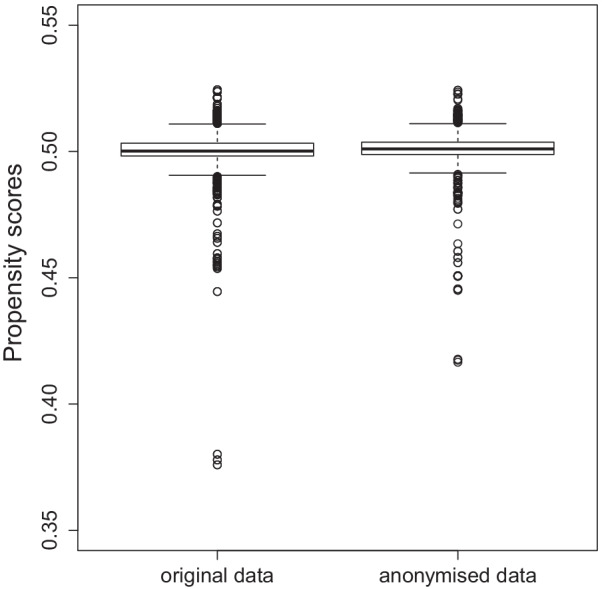
Box plots of the individual propensity scores for each observation of original and anonymised data

The global utility loss is *U* = 0.000117—close to zero—indicating some, but not much, loss of information. Looking individually at the propensity scores for each observation, most are very close to 0.5 (see Fig. 3).

The variable-specific information loss is $\delta _{Age}$ = 0.0114 and $\delta _{Fare}$ = 0.0473 which indicate that around 1.1% and 4.7% of the variability of the anonymised variables *Age* and *Fare* respectively is due to the anonymisation.

**Fig. 4:**
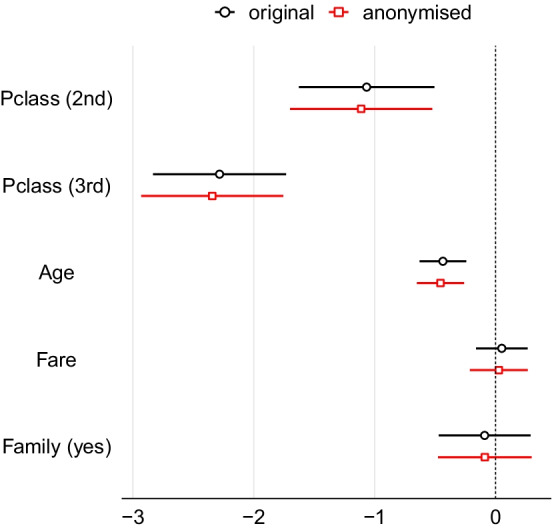
Standardised coefficients and their 95% confidence intervals of the logistic regression model that predicts survival of the Titanic passengers. Black colour denotes the estimates of the model applied to the original data and red colour denotes the estimates of the model applied to the anonymised data. For ease of presentation, the intercept coefficient of both models is not displayed in the plots

We then fit a logistic regression model with the variable *Survived* as the outcome and *Pclass*, *Sex*, *Age*, *Fare* and *Family* as covariates. We do this twice: here we have the luxury of being able to apply it to the original data as well as the anonymised data to compare the similarity of the estimates. The estimated coefficients of the model are shown in Table 3. We observe that the estimated coefficients obtained by the regression model applied to anonymised data are very close to those estimated using the original data. The standardised differences between the coefficients of the model applied to original and anonymised data are also given in Table 3. The confidence intervals of the regression coefficients from the original and anonymised data are also overlapping; see Fig. 4.

**Table 3:** Estimated coefficients of the logistic regression model predicting survival of Titanic passengers using the original (left) and the anonymised (right) data

Coefficient	Original data				Anonymised data				Std. difference
	Estimate	Std. Err.	z value	Pr($>|z|$)	Estimate	Std. Err.	z value	Pr($>|z|$)	
Intercept	3.519	0.437	8.054	8.00e–16	3.615	0.454	7.972	1.56e–15	0.220
Pclass (2nd)	− 1.067	0.286	− 3.732	0.0002	− 1.112	0.300	− 3.707	0.0002	0.159
Pclass (3rd)	− 2.283	0.281	− 8.135	4.12e–16	− 2.343	0.300	− 7.822	5.21e–15	0.216
Sex (male)	− 2.628	0.194	− 13.527	< 2e–16	− 2.625	0.194	− 13.508	< 2e–16	0.012
Age	− 0.033	0.008	− 4.437	9.13e–06	− 0.035	0.008	− 4.586	4.53e–06	0.205
Fare	0.001	0.002	0.466	0.641	0.001	0.002	0.217	0.829	0.223
Family (yes)	− 0.091	0.194	− 0.471	0.638	− 0.089	0.197	− 0.452	0.651	0.010

### The 1958 National Child Development Study

Our second application is to a sample dataset of participants in the National Child Development Study (NCDS), also known as the 1958 Birth Cohort. The NCDS follows the lives of 17,415 people born in England, Scotland and Wales in a single week in 1958 [25]. The data consist of a set of risk factors and phenotypic variables from the 1958 Birth Cohort harmonised for the BioSHaRE-EU Healthy Obese Project, designed to examine the consequences of healthy obesity across several European biobanks and large-scale cohort studies [26]. The sample dataset includes 99 variables for 1469 individuals, and a unique ID. This sample dataset was collected when the birth cohort’s participants were 45 years old.

For the purpose of this paper, we select four continuous and two categorical variables to anonymise and analyse. The continuous variables are the fasting glucose level; the high-density lipoprotein (HDL) cholesterol; and the height and the weight of each participant. The categorical variables are gender (0 = Male and 1 = Female) and a binary variable indicating current cigarette smoking status (0 = Not a current cigarette smoker and 1 = Current cigarette smoker). The dataset has missing values and to avoid imputation this time we select only the complete cases which gives us data on 1211 individuals.

#### Anonymisation

We follow the five steps of anonymisation for the four continuous variables. For step (2)—stratification—we note that the two binary categorical variables form four possible strata: (a) 492 males are not smokers ($\sim$40.6% of the data), (b) 111 males are smokers ($\sim$9.2% of the data), (c) 468 females are not smokers ($\sim$38.6% of the data) and (d) 140 females are smokers ($\sim$11.6% of the data). The minimum stratum has 111 records, confirming that the data are 3-anonymous. We follow the steps of the anonymisation procedure to obfuscate the four continuous variables, using *k* = 3.

#### Disclosure risk and utility loss

**Fig. 5:**
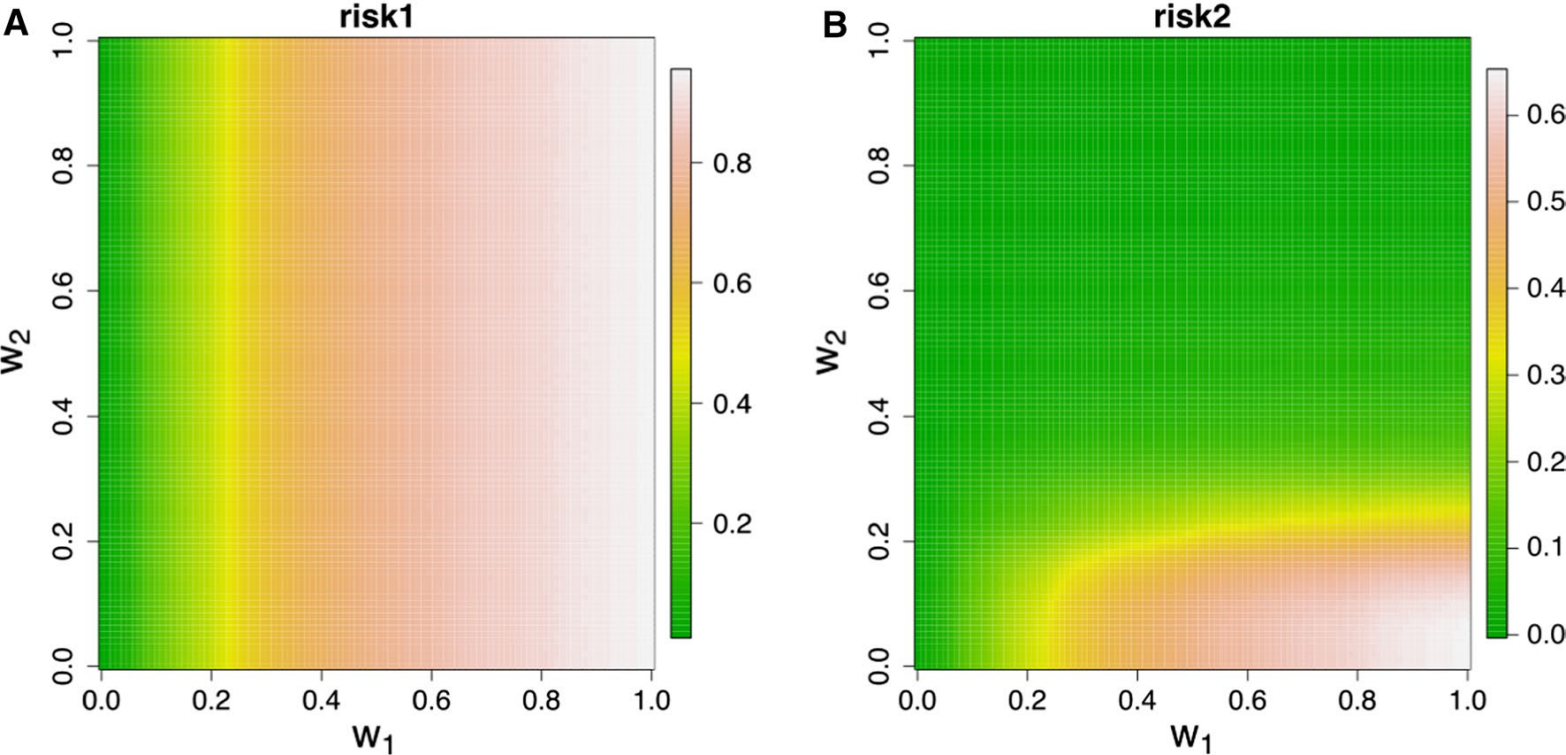
The disclosure risks (**A** risk1 and **B** risk2) estimated by the robust Mahalanobis distance-based metric for different values of the parameters $w_1$ and $w_2$

Once the (continuous) data have been anonymised, we calculate the information loss and disclosure risk metrics. The disclosure risks as estimated by the robust Mahalanobis distance metric with $w_1$ = 0.01 and $w_2$ = 0.05, are *risk1* = 0.0140 and *risk2* = 0.0099. This implies that around 17 of the 1211 observations (1.4%) are risky and 12 observations (0.99%) are potentially unsafe. We have also conducted a sensitivity analysis for the parameters $w_1$ and $w_2$. Figure 5A illustrates that when $w_1$ increases the *risk1* metric also increases as the intervals around the original observations become bigger and there is higher frequency of anonymised data points falling into those intervals. The *risk1* metric is independent of the $w_2$ parameter. Figure 5B shows that when $w_2$ increases, *risk2* decreases because more risky observations fall within bigger intervals thus obscuring the risky data points.

The global information loss of the anonymised data as measured by the summary of propensity scores is *U* = 0.00005: a low score suggesting that there is little in the way of overall loss of utility in anonymising the data. The variable-specific utility losses are $\delta _{Glucose}$ = 0.0466, $\delta _{HDL}$ = 0.0229, $\delta _{Height}$ = 0.0296 and $\delta _{Weight}$ = 0.0247 indicating that less than 5% of the variability of any of the anonymised variables is due to the anonymisation process itself.

**Fig. 6:**
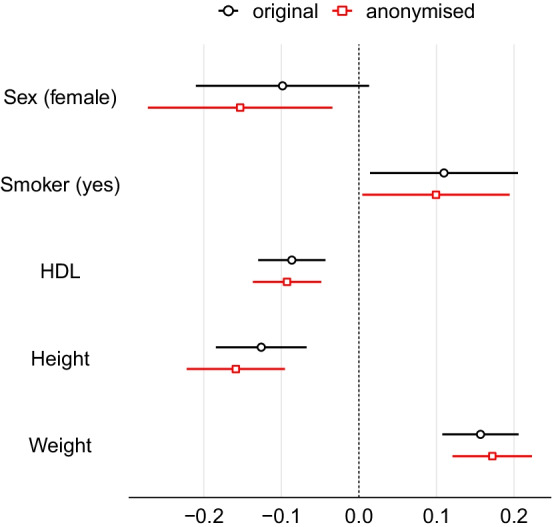
Standardised coefficients and their 95% confidence intervals of the linear regression model that predicts fasting glucose levels of participants in the 1958 Birth Cohort. Black colour denotes the estimates of the model applied to the original data and red colour denotes the estimates of the model applied to the anonymised data. For ease of presentation, the intercept coefficient of both models is not displayed in the plots

We then apply a linear regression model to predict the fasting glucose level, with *Sex*, *Smoker*, *HDL*, *Height* and *Weight* as covariates. The estimated parameters of the model fitted to the original and the anonymised data are shown in Table 4. The estimated coefficients using the anonymised data are very close to the estimated coefficients using the original data. This yields small standardised differences between the coefficients of the model applied to original and anonymised data, which are also shown in Table 4. The similarity is perhaps easier to see in Fig. 6 in the overlapping of the confidence intervals.

**Table 4:** Estimated coefficients of the linear regression model predicting fasting glucose level of participants in the National Child Development Study using the original (left) and the anonymised (right) data

Coefficient	Original data				Anonymised data				Std. difference
	Estimate	Std. Err.	t value	Pr($>|t|$)	Estimate	Std. Err.	t value	Pr($>|t|$)	
Intercept	6.430	0.527	12.211	<2e–16	6.982	0.564	12.379	<2e–16	1.048
Sex (female)	− 0.098	0.057	− 1.729	0.084	− 0.153	0.061	− 2.524	0.012	0.959
Smoker (yes)	0.110	0.049	2.253	0.024	0.099	0.048	2.054	0.040	0.212
HDL	− 0.229	0.058	− 3.924	9.19e–05	− 0.245	0.059	− 4.119	4.06e–05	0.278
Height	− 0.013	0.003	− 4.225	2.57e–05	− 0.017	0.003	− 4.916	1.01e–06	1.10
Weight	0.010	0.002	6.265	5.18e–10	0.011	0.002	6.589	6.59e–11	0.606

### Sensitivity analysis

We perform a sensitivity analysis to examine the effect of the choice of *k* (which dictates the number of nearest neighbours we seek for each observation) and the effect of the sample size on the disclosure risk and information loss of the non-stochastic anonymised data. We base the generation of synthetic data on 1958 Birth Cohort data in the previous section, using the observed distributions of the variables from the complete cases.

#### Choice of *k*

We set the size of the sample to 500 records. The four possible strata are created with the same proportions as seen in the real data, yielding 203 non smoking males (40.6% of 500), a stratum with 46 male smokers (9.2% of 500), a stratum with 193 non smoking females (38.6% of 500) and a stratum with 58 female smokers (11.6% of 500). For each stratum we simulate the four continuous variables. To simulate these non-normal data we use the Vale and Maurelli method [37]. The simulation of the non normal multivariate distribution is based on the observed mean, skewness and kurtosis of each continuous variable in the original data, as well as all bivariate covariances.

For each simulated dataset we apply the deterministic anonymisation and calculate the disclosure risks and utility loss metrics. We apply the analysis for a *k* of 3, 5, 7, 10, 15, 20, 25, 30, 35, 40, and 46. The value of 46 is the maximum value that we can use as this is the number of records in the smallest stratum of the simulated data. In that case all data belonging to that stratum are replaced by the same centroid (and we would expect substantial loss of information).

**Fig. 7:**
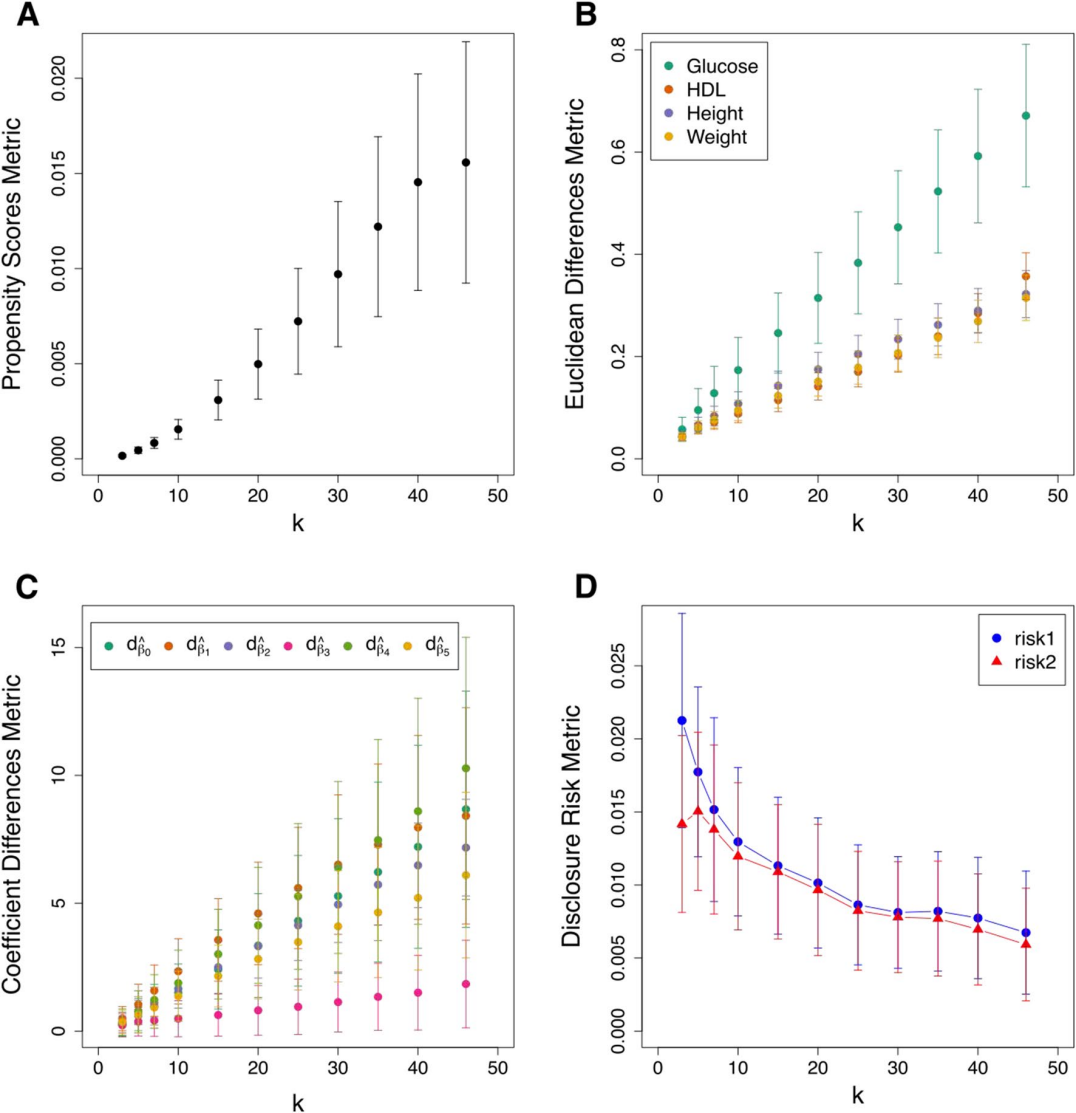
The effect of the number of nearest neighbours (parameter *k*) on utility loss and disclosure risk of non-stochastic anonymised data. **A** The dataset-specific utility loss as measured by the summary statistic *U* of propensity scores. **B** The variables-specific utility loss as measured by the Euclidean distance-based metric. **C** The analysis-specific information loss as measured by the standardised difference of regression model coefficients. **D** The robust Mahalanobis distance-based disclosure risks. Each point and error bar in the four panels indicates the mean plus minus one standard deviation of the metrics across 100 generated synthetic samples of 500 individual-level records each

For each value of *k* we generate 100 datasets, which will differ in their values of the continuous variables. The results of the mean of the metrics considered across all 100 datasets for each *k* are shown in Fig. 7. Figure 7A shows the increase in the global utility loss as measured by the propensity scores based metric as *k* increases; Fig. 7B shows the increase of the standardised Euclidean distances between original and anonymised data points as *k* increases; Fig. 7C presents the increase of the standardised difference between estimated regression coefficients with the original and anonymised data, as *k* increases; and Fig. 7D shows the reduction in the disclosure risk as *k* increases. All three measures of utility show information loss as we increase *k*, which is to be expected. Unsurprisingly they also show the typical trade-off between disclosure risk and utility loss: lower values of *k* are preferable in terms of utility but this increases the risk of disclosure.

#### Sample size

Our second sensitivity analysis looks at the effect of sample size on the disclosure risk and information loss when using anonymised data. This analysis also accounts for the impact of the size of the minimum stratum: a smaller sample size will yield smaller minimum stratum.

For this analysis we fix the value of *k* to 5 and simulate sample sizes of 50, 100, 200, 500, 1000, 1500, 2000, 2500, and 3000. We follow the same data generating mechanism as described in the previous section, but this time with fixed *k* and varying sample sizes. With just 50 observations, the four strata have 20, 5, 19 and 6 records respectively. Setting *k* = 5 is therefore permissible, though we should expect major loss of information in this case especially for the two smallest strata.

**Fig. 8:**
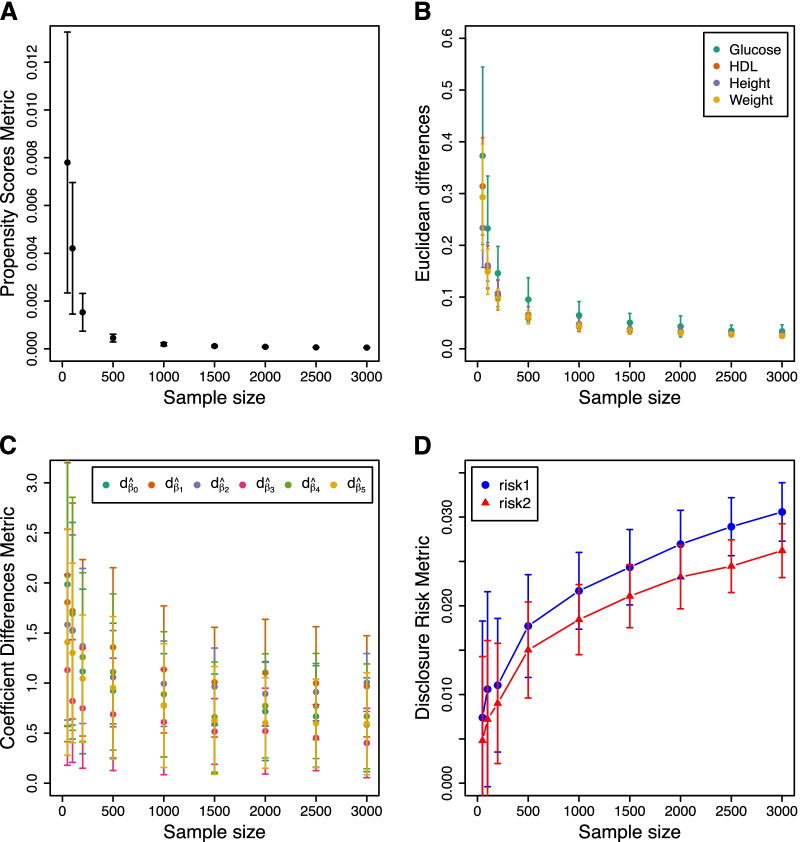
The effect of the sample size on utility loss and disclosure risk of non-stochastic anonymised data. **A** The dataset-specific utility loss as measured by the summary statistic *U* of propensity scores. **B** The variables-specific utility loss as measured by the Euclidean distance-based metric. **C** The analysis-specific information loss as measured by the standardised difference of regression model coefficients. **D** The robust Mahalanobis distance-based disclosure risks. Each point and error bar in the four panels indicates the mean plus minus one standard deviation of the metrics across 100 simulations with constant *k* = 5

For each sample size we generate 100 datasets (these will differ in terms of their ‘original’ continuous variables). The results from anonymising these 100 datasets, for each sample size considered, are shown in Fig. 8. Unsurprisingly all three metrics for utility improve as we increase sample size while the disclosure risk also increases as we increase sample size. The reason for this lies with the algorithm for anonymisation: as the sample size increases, so do the number of observations lying close to some other observation. So, for fixed *k*, the *k* nearest neighbours will potentially be closer compared to the situation where the dataset is much smaller. This results in using centroids that are closer to the original observation, which subsequently increases the risk. However, this needs to be traded-off with the idea that if an observation has ‘neighbours’ that are relatively close, then the risk of actual disclosure is reduced.

## Discussion

The evolution of data science exploits the availability of large, granular datasets with detailed information, and enhances the advancement of technologies, methods and applications that are used to translate such data into tools that can benefit society. However, with great potential in using data comes major challenges. One of those challenges is the restricted access to personal data especially in disciplines where data include sensitive information (from healthcare to banking).

The demand for access to such personal data has fuelled the exploration and development of several tools and approaches that allow data to be shared and analysed within secure frameworks that preserve privacy and maintain data subjects’ confidentiality. Deciding on the framework for mitigating disclosure risk of personal data is the responsibility of data custodians, who must make decisions based on a careful assessment of real risks that are typically context dependent. Suggested solutions have different strengths and weaknesses, and the final choice of the optimal method should depend on a rigorous assessment of the real risk of disclosure and the gravity of it occurring, versus the magnitude of the drawbacks of applying privacy protection including formal evaluation of potential information loss that must be tailored to the specific context that applies. In many cases simple data obfuscation through suppression and aggregation may be enough to mitigate the risk of disclosure while in other contexts more complex techniques might be required.

In this paper we propose a new deterministic approach for anonymising personal data that increases the uncertainty of data re-identification while limiting the loss of utility. This strategy therefore allows informative statistical analysis, but relies on having at least one continuous or numeric variable in the dataset. The method stratifies an underlying dataset by all possible combinations of the levels of categorical variables. The values of continuous variables are then replaced by taking into consideration the observation’s $(k-1)$ nearest neighbours. This re-distribution of the continuous variables in a dataset preserves their spatial correlations.

Our demonstration of the algorithm in “Applications” section shows that the procedure preserves the structure of the data well in cases where the required analysis is a regression model. The sensitivity analysis revealed the risk of disclosure and the utility loss are both dependent on sample size—which also tells us indirectly that it is dependent on the size of the strata—and also on our choice of *k*.

The final choice of the parameters in *k*-anonymity and *k*-nearest neighbours depends on a rigorous assessment of the real risks of disclosure and the magnitude of the information loss generated by applying the anonymisation process. In general, this association is highly context dependent, and therefore the selection of the parameters must be specified based on each specific data situation. In many cases data protection through small values of the parameters in *k*-anonymity and *k*-nearest neighbours may be enough to mitigate the risk of disclosure and preserve data utility, while in other contexts bigger values may be preferable [24].

This procedure has several advantages over other methods of anonymisation. The most important is that we introduce three layers of protection. The first layer occurs due to stratification: in cases where the strata themselves do not give rise to well-defined clusters of observations (which is often the case for real data), the search for the nearest neighbours *within* a stratum obfuscates the actual proximity of the data. That is, the nearest neighbours of a data point *within* a stratum might be different to its nearest neighbours if we consider the *entire* dataset. The nearest neighbours within a stratum will be at least as far as an observation’s nearest neighbours globally, so this increases the disturbance of each data point. This effect is greater for ‘outliers’ than for data points within a mass of observations. For example, if the $k-1$ nearest neighbours of an unusual observation (that is distant from the majority of the other observations in the dataset) are located within the cloud of the mass of the data, then the corresponding centroid is located closer to the data mass in contrast to the original outlier point. This does not mean that the anonymised data lie exclusively within the convex hull of the original data: the scaling that occurs in step (5) of the algorithm ensures that it is possible to obtain anonymised data that are outside this region.

The second layer of protection is the one to one replacement of each data point with the centroid of the point itself and its $(k-1)$ nearest neighbours. Note that any value of $k>2$ makes the calculation of the exact original values almost impossible (with *k* = 2, a malicious ‘attacker’ could potentially disclose the exact value of an observation but only if they knew the centroid replacing a data point, and the mean and the variance of the three original observations that created the centroid). The optimal value for *k* is context-specific to minimise loss of utility as much as possible while ensuring a tolerable level of risk disclosure. A discussion on the optimal value of *k* could be linked to a wider review on the threshold rules used in other privacy protection techniques; for example, a similar disclosure control applied to a 2-dimensional contingency table where if at least one cell has fewer counts than an agreed threshold, the information in the rest of the table is not released. Simple threshold rules like these for the proposed algorithm in this paper would help to ensure that the anonymised dataset contained no obviously disclosive information. Of course, such thresholds could never universally guarantee safety.

The third layer of protection is a result of the scaling of the centroids introduced in step (5) that introduces extra uncertainty to the anonymised data. The scaling is applied to the entire dataset and not within each stratum separately and shifts the centroid back to the observed variance of each variable. The direction in which the scaling factor shifts each coordinate of a centroid depends on its position in relation to the origin, ${\mathbf {0}}=(0,\ldots ,0)$. For example, in a 2D-space the *x*-coordinate of a centroid is multiplied by the scaling factor of variable *x* and the *y*-coordinate of the centroid is multiplied by the scaling factor of the variable *y*. Therefore, the direction of the shift of each centroid due to scaling depends on the quadrant within which the centroid exists around the origin, (0, 0).

Another advantage of the proposed method is that its non-stochastic behaviour ensures that it generates the same anonymised data for any given underlying dataset and a fixed value of *k*. This gives data managers full control of data relocation at each step of the anonymisation procedure (in contrast to stochastic perturbations) and therefore based on their risk assessments to choose the optimal value of *k* for their specific situation. It also prevents disclosure attacks based on generating multiple datasets and then using the law of large numbers to obtain the underlying values of the data before perturbation.

No anonymisation procedure provides a perfect solution, and some limitations with the proposed procedure are evident. The first is how we deal with missing data (note that in other methods, e.g. purely synthetic data, this is not a problem). The decision is related to the sample size of the data and the information loss if we reduce the dataset to its complete case format. If the decision is to keep the sample size unchanged then we will handle missing values in continuous variables differently to those missing values in categorical variables. For missing values in continuous variables we can either use sophisticated imputation methods or we can simply replace the empty cells with the median (or the mean) of the non-missing values within each column separately and independently from the others. For the categorical variables we can either impute missing values using a binomial distribution fitted to the non-missing values or we can consider missing values as another level of the categories of each variable.

Aside from issues with missing data, note that *k*-anonymity should be applied across all strata and in some cases this is an advantage: it adds an extra level of protection to the categorical variables. However, in big datasets with many categorical variables, or in smaller datasets where categorical variables have many levels, achieving *k*-anonymity might be challenging. Ways around these issues include combining categories to reduce the number of strata, or in extreme cases removing one or more categorical variable may be necessary. Another limitation is the potential instability of the *k*-NN classification in high-dimensional data [38–41]. This instability can be reflected in both the performance of the algorithm and on the level of the loss of utility on the anonymised data. In such cases, the data custodian might decide to use the algorithm in a subset of variables (like a pseudonymisation process), especially on those associated with higher disclosure risk (e.g. direct and indirect identifiers).

## Conclusion

In this paper, we propose a deterministic algorithm for data anonymisation, as a possible solution to eliminate some of the barriers to data access, and in doing so ensure that the scientific principles of transparency and reproducibility are maintained beyond the immediate research domain. The deterministic approach can be used across different domains where data are viewed as sensitive either because of concerns relating to information governance and/or data ethics, or because of their value in terms of intellectual property. The proposed approach would be of interest to a wide range of data stakeholders: not just data custodians, but also data analysts, study participants and the general public who all have an interest in ensuring that data are used as widely as possible in ways that are of value to society and yet protect their confidentiality. Unlike many other privacy preserving methods, the procedure of deterministic anonymisation still allows researchers access to informative data that retain the characteristics of the original data so that there is no restriction on how the data can be analysed. Therefore, this approach has the potential to enhance the discoverability and utility of individual-level data across all disciplines where data are sensitive. The use of such approach is essential to support the collection and appropriate use of personal data, to maintain the trust of participants joining research studies, and to enhance the compliance with the increasingly robust frameworks. These underpin the governance and ethico-legal stipulations and regulations oversight management, process and use of sensitive personal data.

## Data Availability

This work made use of data and samples generated by the 1958 Birth Cohort (NCDS), which is managed by the Centre for Longitudinal Studies at the UCL Institute of Education, funded by the Economic and Social Research Council (Grant No. ES/M001660/1). Access to these resources was enabled via the Wellcome Trust & MRC: 58FORWARDS Grant [108439/Z/15/Z] (The 1958 Birth Cohort: Fostering new Opportunities for Research via Wider Access to Reliable Data and Samples). Before 2015 biomedical resources were maintained under the Wellcome Trust and Medical Research Council 58READIE Project (Grant Nos. WT095219MA and G1001799). In addition, this work made use of a sample of Titanic passengers data which is publicly available on the webpage https://www.kaggle.com/c/titanic/data.
